# Identification of Key Genes and Pathways in Post-traumatic Stress Disorder Using Microarray Analysis

**DOI:** 10.3389/fpsyg.2019.00302

**Published:** 2019-02-27

**Authors:** Yaoyao Bian, Lili Yang, Min Zhao, Zhengjun Li, Yuying Xu, Guilian Zhou, Wenlin Li, Li Zeng

**Affiliations:** ^1^School of Nursing, Nanjing University of Chinese Medicine, Nanjing, China; ^2^School of First Clinical Medicine, Nanjing University of Chinese Medicine, Nanjing, China; ^3^Jingwen Library, Nanjing University of Chinese Medicine, Nanjing, China; ^4^Management School, Lancaster University, Lancaster, United Kingdom

**Keywords:** PTSD, bioinformatics analysis, microarray analysis, key genes, key pathways

## Abstract

**Introduction:** Post-traumatic stress disorder (PTSD) is characterized by impaired fear extinction, excessive anxiety, and depression. However, the potential pathogenesis and cause of PTSD are not fully understood. Hence, the purpose of this study was to identify key genes and pathway involved in PTSD and reveal underlying molecular mechanisms by using bioinformatics analysis.

**Methods:** The mRNA microarray expression profile dataset was retrieved and downloaded from the Gene Expression Omnibus (GEO) database. The differentially expressed genes (DEGs) were screened using GEO2R. Gene ontology (GO) was used for gene function annotations and Kyoto Encyclopedia of Genes and Genomes (KEGG) pathway was performed for enrichment analysis. Subsequently, protein–protein interaction (PPI) network and module analysis by the plugin MCODE were mapped by Cytoscape software. Finally, these key genes were verified in stress-exposed models by Real-Time quantitative (qRT-PCR). In addition, we performed text mining among the key genes and pathway with PTSD by using COREMINE.

**Results:** A total of 1004 DEGs were identified. Gene functional annotations and enrichment analysis indicated that the most associated pathway was closely related to the Wnt signaling pathway. Using PPI network and module analysis, we identified a group of “seed” genes. These genes were further verified by qRT-PCR. In addition, text mining indicated that the altered CYP1A2, SYT1, and NLGN1 affecting PTSD might work via the Wnt signaling pathway.

**Conclusion:** By using bioinformatics analysis, we identified a number of genes and relevant pathway which may represent key mechanisms associated with PTSD. However, these findings require verification in future experimental studies.

## Introduction

Post-traumatic stress disorder (PTSD) is defined as affective trauma-related or stressor-related disorder exposed to single/episodic, direct/indirect, or acute/chronic events (Ronzoni et al., 2016; Kim et al., 2018). It manifests with a multitude of clinically significant symptoms including avoidance when re-living the traumatic event, disturbing when reminding the flashbacks and hyper vigilance often last for at least 1 month after the occurrence of the event (Dennis et al., 2016; Eustache et al., 2017).

More and more literatures on the psychopathological consequences of trauma exposure or life-threatening events have focused upon PTSD. The estimated prevalence of PTSD performed in six countries ranged from 3.1 to 61.6% according to the International Consortium to Predict PTSD (ICPP) project (Qi et al., 2018). In a recent meta-analysis of 27 studies including 30,878 ambulance personnel, a 11% prevalence rate of PTSD was found, appearing to be particularity at high risk (Petrie et al., 2018). Similary, in China, the prevalence was reported to range from 1.3 to 62.8% after 2008 *Wenchuan* earthquake (Hong and Efferth, 2016). PTSD can not only cause multisystem disorders with comorbidities both physically and mentally, but also it can lead to a number of negative social consequences such as suicide or violence tendencies. It has brought a significant personal and societal burden.

To date, various researches suggested that pathogenesis of PTSD was associated with autonomic nervous system (ANS), hypothalamic-pituitary-adrenal (HPA) axis, neural circuits and immune system. The underlying pathogenesis of PTSD remains incompletely unknown. Therefore, it is promoting the need to develop a further identifying the etiological factors, molecular mechanisms, and pathways of PTSD to discover novel diagnostic and treatment strategies for PTSD.

Fortunately, with the advances of sequencing and high-throughput DNA microarray analyses, numerous genes and pathways have been demonstrated to be correlated with the genesis and progression of PTSD. For example, Kilaru et al. (2016) found that Neuroligin 1 (NLGN1) might participate in synaptic plasticity, which further suggesting a significant association between Neuroligin 1 (NLGN1) and PTSD. Maheu and Ressler (2017) found that Wnt protein was related to fear- and stress-related disorder. Moreover, various genes, i.e., FK506 Binding Protein 5 (FKBP5) (Young et al., 2015), Dicer 1, Ribonuclease III (DICER1) (Wingo et al., 2015), and Dopamine D2 receptor (DRD2) (Duan et al., 2015) were reported to participate in cellular pathway of PTSD. Also, various gene pathways have been shown to be important, such as mTOR pathway (Oh et al., 2018), ERK pathway (Xiang et al., 2017), and Akt/GSK-3β signaling pathway (Chen et al., 2015), etc. Therefore, identifying differentially expressed genes (DEGs) and pathways, elucidating the interactions network among them, are essential for PTSD.

In this study, we retrieved dataset of mRNA expression microarrays from Gene Expression Omnibus (GEO), and identified a subset of genes as biomarkers in PTSD by using bioinformatics analysis. In addition, several candidate targets for following experimental research were performed. This finding can further help us understand underlying pathogenesis associated with PTSD, and provide initial evidence for future study on potential mechanisms of PTSD.

## Materials and Methods

### Data Acquisition and DEGs Identification

The mRNA microarray expression profile dataset was retrieved and downloaded from the GEO database (available online: http://www.ncbi.nlm.nih.gov/geo). After screening, GSE68077 was obtained for our analysis. The platform for GSE68077 was GPL7202, Agilent-014868 Whole Mouse Genome Microarray 4x44K G4122F (Muhie et al., 2017). This dataset consists of 346 groups including brain transcriptome profiles in mouse model simulating features of PTSD and transcriptome profiling of spleen, blood, and hemi-brain of social stressed C57BL/6 mice exhibiting PTSD like features. The C57BL/6 mice were exposed to SJL aggressor mice for periods of 5 or 10 days (6 h each day) to induce anxiety/stress which parallels to PTSD in human. Organs, blood, and brain regions were collected after 1 day and 1.5 weeks following 5 days trauma-exposed, and 1 day and 6 weeks following 10 days trauma-exposed. In current study, the microarray data of hippocampus 6 weeks after 10 days social stressed was collected for analysis. DEGs were screened using GEO2R, an online analytical tool available in GEO. The |logFC| > 1 and *P* < 0.05 were used as the cutoff values for significantly DEGs. Limma package in the Bioconductor package (available online: http://www.bioconductor.org/) was used for gene differential expression analysis.

### Functional and Pathway Enrichment Analysis of DEGs

Gene ontology, a method for annotating genes, was performed to identify potential biological processes, i.e., biological processes (BP), cellular component (CC), and molecular function (MF). The Kyoto Encyclopedia of Genes and Genomes (KEGG) pathway was conducted for presenting the annotation and visualization of gene functions. In addition, both GO enrichment and KEGG pathway analysis were performed using the Database for Annotation, Visualization and Integrated Discovery (DAVID^1^) (Huang et al., 2007) to understand the biological significance of genes, when *P*-values <0.05 was considered as cutoff criterion.

### Protein–Protein Interaction (PPI) Network Construction and Module Analysis

The Search Tool for the Retrieval of Interacting Genes (STRING^2^) (von et al., 2003), an online tool for annotation of protein cellular localization and biological function, was conducted to predict protein–protein interaction (PPI) information. DEGs were mapped to STRING to evaluate the interaction relationships, with a confidence score >0.9 defined as significant, and PPI integrated networks were visualized by Cytoscape software (Shannon et al., 2003). Then, the plug-in Molecular Complex Detection (MCODE) from Cytoscape was applied to screen the modules of PPI network. Finally, Text mining of gene function prediction was conducted by COREMINE^3^ (de Leeuw et al., 2012).

### Experimental Animals

#### Aggressor Mice

Six 6-week-old male SJL albino mice were purchased from Beijing Vital River Laboratory Animal Technology Co., Ltd. The mice were initially weighted 30–35 g. And these mice were individually housed in the plexiglas cages (48 cm × 27 cm, height 20 cm) with free water and food in a 12-h light/dark cycle (lights on 6 AM to 6 PM) for 30 days. The temperature was controlled at 22 ± 2°C and relative humidity was kept at 30–60%. In the meantime, these mice were trained to induce aggressiveness due to isolation (Hammamieh et al., 2012).

#### Subject Mice

Twelve male C57BL/6N mice (8 weeks old weighing 18 ± 2 g) were provided by the Qinglongshan Experimental Animal Breeding Farm (Nanjing, China). Animal were randomly into two groups: control group and aggressor-exposed (Agg-E) group. Two groups were housed at room temperature 22 ± 2°C with standard condition of 12 h light and 12 h darkness. All mice had plenty food and water freely. The experiments were conducted under the approval of Laboratory Animal Management Committee of Nanjing University of Chinese Medicine (approval ID: 201810A043). All procedures were compliant with the Guidelines of Accommodation and Care for Animals formulated by the Chinese Convention for the protection of vertebrate animals used for experimental and other scientific purposes.

#### Aggressor Exposure

**FIGURE 1:**
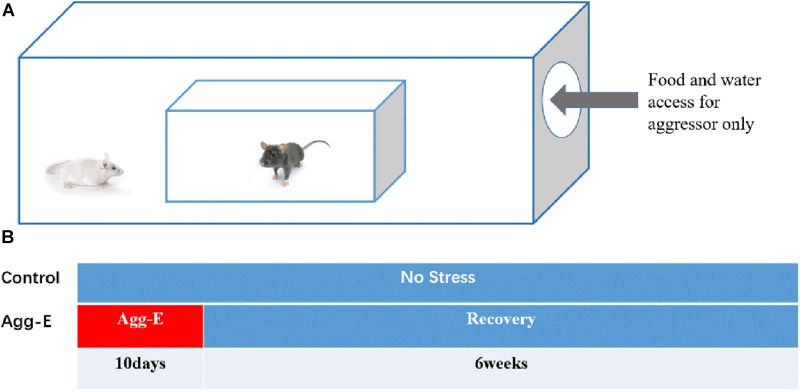
**(A)** Cage-within-cage configuration for aggressor exposure. **(B)** The protocol timeline of single-housed home cage (10 consecutive days with aggressor exposure and 6 weeks rest).

According to a modified “cage-within-cage resident-intruder” protocol (Porsolt et al., 1977), Agg-E mice were placed in a wire mesh cage that was kept inside the aggressor’s large home cage for 6 h. The size of the above cages were 17 cm × 14 cm × 8 cm and 50 cm × 30 cm × 20 cm, respectively, as shown in Figure 1A. Similary, the control mice were placed in the same environment without being exposed by aggressor mice. During each 6 h session, the aggressor mice were given plentiful food and water, while the Agg-E mice and control mice were deprived of food and water. In addition, at one to three random times, Agg-E mice were exposed directly to the aggressor mouse for 1 min or until 10 bites. At the end of each session, the control and Agg-E mice were returned to their home cage where the food and water were plentiful. A total of 10 consecutive days were repeated. After 10 days with aggressor exposure, mice were housed about 6 weeks with food and water *ad libitum*, as shown in the protocol timeline (Figure 1B).

### Forced Swimming Test (FST), Tail Suspension Test (TST), and Open-Field Test (OFT)

Behavioral tests were performed with Smart3.0 tracking software (Panlab). The subject mice were randomly assigned to two groups (*n* = 6/group). After 10 days stressed exposure directly or indirectly, the Agg-E group was fed for 6 weeks for recovery. The forced swimming test (FST) and the tail suspension test (TST) were performed to identify the depression-like behavior of the mice in the Agg-E group. Both FST and TST were performed according to previous studies (Porsolt et al., 1977; Steru et al., 1985). Mice were forced to swim in 20 cm water temperature 25 ± 1°C in 2000 ml glass beaker for 5 min. Immobility time in the last 4 min were measured in the FST test. After FST test, mice were allowed to have a rest for 24 h and then hanged for 6 min. Immobility time in the last 4 min were recorded in the TST test. The open-field test (OFT) is used to evaluate the state of autonomic movement, aiming to identify agitation and pathological behavior. The device is a square bucket with a bottom. The bottom surface is divided into 25 small squares. The movement of each mouse was recorded for 5 min by a video camera.

### RNA Extraction and qRT-PCR

Hippocampus tissue of both control and Agg-E group were extracted total RNA using TRIzol reagent (Invitrogen), following the manufacturer’s instructions. RNA quality and quantity were measured by Nucleic Acid Protein Detector. cDNA was synthesized with total RNA using the First Strand cDNA Synthesis Kit (TaKaRa). The relative expression level of mRNAs was performed by using 2^−ΔΔCT^ analysis method. The primers used were as follows (Table 1). GAPDH expression served as internal control.

**Table 1:** Primers used for qRT-PCR.

Gene	Primer sequence (5′–3′)
GRM2-Forward	CTGTCTCTCTATCTCTCTGC
GRM2-Reverse	TGTGTGTGTGTAACATGATGG
CYP1A2-Forward	AGTACATCTCCTTAGCCCCAG
CYP1A2-Reverse	GGTCCGGGTGGATTCTTCAG
CDH5-Forward	CACTGCTTTGGGAGCCTTC
CDH5-Reverse	GGGGCAGCGATTCATTTTTCT
SF1-Forward	CATGGCAGCAAAGATCCCTC
SF1-Reverse	AAGTCCTCACTCTCATGGCTC
EDN3-Forward	CCCTGGTGAGAGGATTGTGTC
EDN3-Reverse	CCTTGTCCTTGTAAGTGAAGCAC
SYT1-Forward	CTGTCACCACTGTTGCGAC
SYT1-Reverse	GGCAATGGGATTTTATGCAGTTC
CAB39L-Forward	CAAAACGCAGCCTATCGTGGA
CAB39L-Reverse	CTCGTCGTCTGTCCTTTCTTTC
NLGN1-Forward	GGTACTTGGCTTCTTGAGCAC
NLGN1-Reverse	CTTGTTTGGGTATAAAGCCTCCA
GAPDH-Forward	AGGTCGGTGTGAACGGATTTG
GAPDH-Reverse	TGTAGACCATGTAGTTGAGGTCA

### Statistical Analyses

The data of FST, TST and OFT were presented as the mean ± standard deviation (SD) using SPSS19.0 statistical analysis software. The data were analyzed using *t*-test. ^∗^*P* < 0.05 were considered statistically significant.

## Results

### Identification of DEGs

**FIGURE 2:**
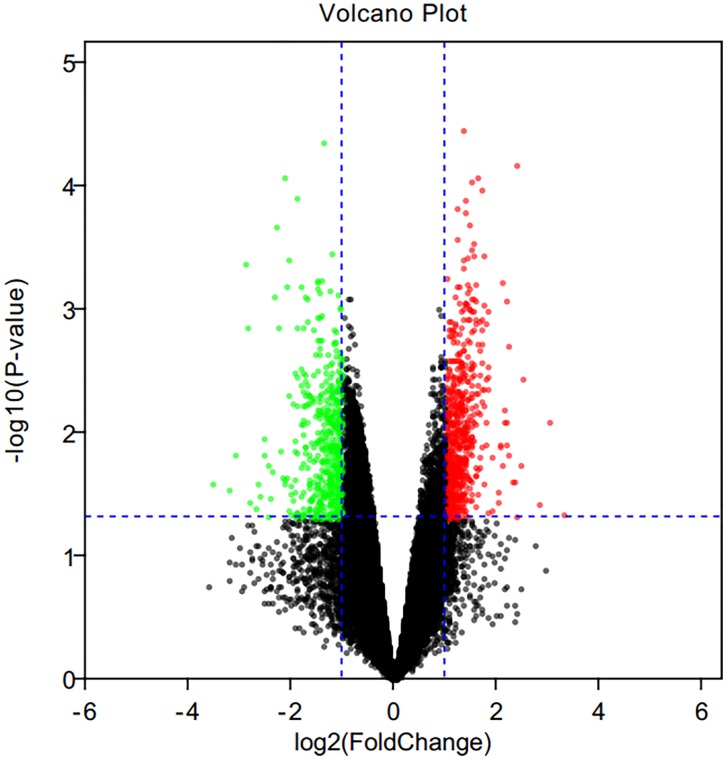
Volcano plot of differential expression genes. Red points as up-regulated genes, green plots as down-regulated genes, and black plots as genes with no significant difference.

The mRNA expression profile datasets in hippocampus region consisted of expression data matrix of 41,175 gene probes. Using GEO2R, we identified 1004 DEGs consisting of 583 up-regulated DEGs and 421 down-regulated DEGs. The differential expression genes were shown in Figure 2.

### Functional Annotations

The 1004 genes were uploaded to DAVID database for biological function assessment, and KEGG pathway enrichment. The results showed that these DEGs were markedly enriched in one of the following 10 biological processes: cellular calcium ion homeostasis, response to calcium ion, complement activation, classical pathway, plasma membrane, negative regulation of cytokine secretion, Wnt signaling pathway, transferase activity, transferring glycosyl groups, cell adhesion, cell junction and calcium ion binding (Table 2). We found that the most significantly enriched pathways were retrograde endocannabinoid signaling, ECM-receptor interaction, complement and coagulation cascades (Table 3). Gene functional annotations and enrichment analyses indicated that the most associated biological function was the Wnt signaling pathway.

**Table 2:** Gene ontology analysis of differentially expressed genes associated with PTSD.

Term	Function	Gene count	*P*-value
GO:0006874	Cellular calcium ion homeostasis	9	<0.01
GO:0051592	Response to calcium ion	8	<0.01
GO:0006958	Complement activation, classical pathway	7	<0.01
GO:0005886	Plasma membrane	237	<0.01
GO:0050710	Negative regulation of cytokine secretion	4	<0.01
GO:0016055	Wnt signaling pathway	15	0.01
GO:0016757	Transferase activity, transferring glycosyl groups	16	0.01
GO:0007155	Cell adhesion	29	0.03
GO:0030054	Cell junction	40	0.03
GO:0005509	Calcium ion binding	40	0.03

**Table 3:** Kyoto Encyclopedia of Genes and Genomes pathway analysis of differentially expressed genes associated with PTSD.

Term	Definition	Gene count	*P*-value
mmu04610	Complement and coagulation cascades	11	<0.01
mmu04512	ECM-receptor interaction	10	0.01
mmu04723	Retrograde endocannabinoid signaling	9	0.04

### Protein and Protein Network

**FIGURE 3:**
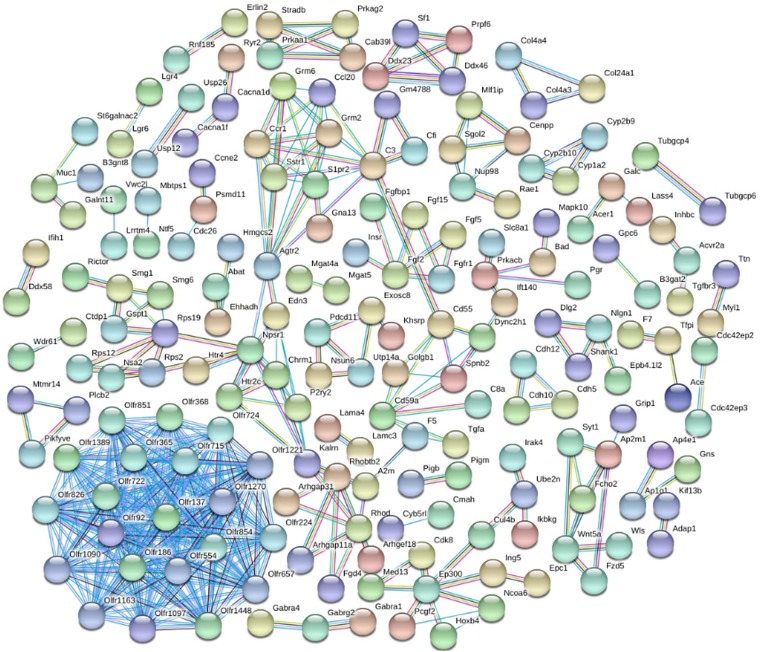
The differential expressed protein–protein interaction network. Proteins were represented with color nodes, and interactions were presented with edges.

In order to mine the PTSD-associated genes, we performed PPI network analysis by STRING database, and 415 PPI pairs were derived (Figure 3), which then underwent analysis by Cytoscape to depict the complex relationship (combined score >0.9). Then, 16 clusters were selected from PPI network using the plugin MCODE. MCODE analysis showed that each cluster had one “seed” gene. The “seed” genes were as follows: Olfr1389, Glutamate Metabotropic Receptor 2 (GRM2), Endothelin 3 (EDN3), Golgin B1 (GOLGB1), Synaptotagmin 1 (SYT1), Splicing Factor 1 (SF1), G1 To S Phase Transition 1 (GSPT1), Calcium Binding Protein 39 Like (CAB39L), Exosome Component 8 (EXOSC8), FGF15, Cadherin 5 (CDH5), Collagen Type XXIV Alpha 1 Chain (COL24A1), Cytochrome P450 Family 1 Subfamily A Member 2 (CYP1A2), Myotubularin Related Protein 14 (MTMR14), Neuroligin 1 (NLGN1), and Shugoshin 2 (SGOL2). We found that the main roles of these genes were in cell junction, cell adhesion molecules (CAMs).

### Verification of “Seed” Genes and Potential Pathway

**FIGURE 4:**
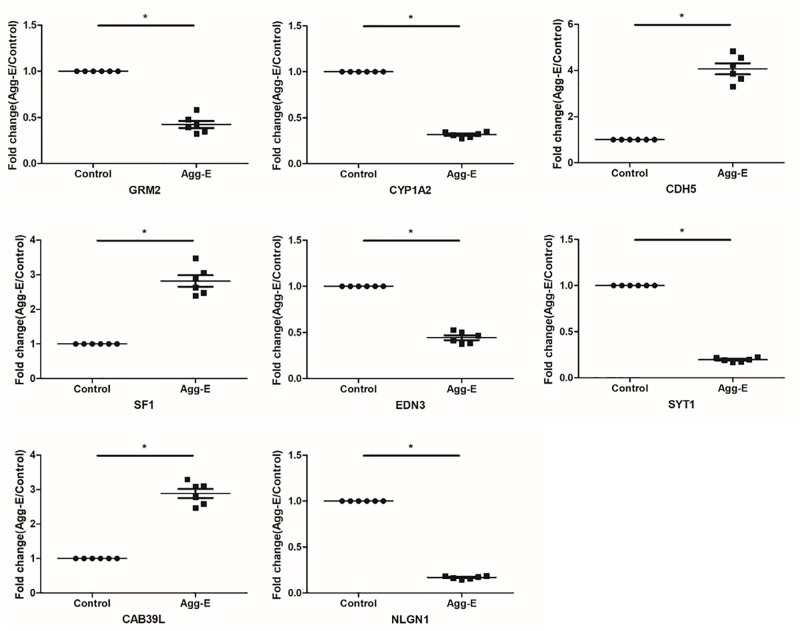
Relative expression level of the “seed” genes in hippocampus region in response to stress exposure. The expression level of mRNAs was conducted using qRT-PCR. Results were shown as mean ± SD, ^∗^*P* < 0.05.

To verify the “seed” genes, we used qRT-PCR to identify the expression level of these differentially expressed mRNAs in hippocampal tissue between two groups (Figure 4). The results of qRT-PCR showed that all “seed” genes, only GRM2, CYP1A2, CDH5, SF1, EDN3, SYT1, CAB39L, and NLGN1 were differentially expressed (*P* < 0.05, Table 4), which were consistent with the microarray datasets. It is suggested that the eight “seed” genes may function as a group, and play a vital role in pathological mechanism to PTSD.

**Table 4:** Relative expression level of eight “seed” genes in hippocampus region.

Gene	Up/down regulation	Fold change (mean ± SD)	*P*-value
GRM2	Down	2.454 ± 0.384	*P* < 0.05
CYP1A2	Down	3.184 ± 0.298	*P* < 0.05
CDH5	Up	4.071 ± 0.579	*P* < 0.05
SF1	Up	2.821 ± 0.407	*P* < 0.05
EDN3	Down	2.288 ± 0.321	*P* < 0.05
SYT1	Down	5.146 ± 0.578	*P* < 0.05
CAB39L	Up	2.887 ± 0.325	*P* < 0.05
NLGN1	Down	5.951 ± 0.596	*P* < 0.05

### Text Mining of Genes and Pathway With PTSD

**FIGURE 5:**
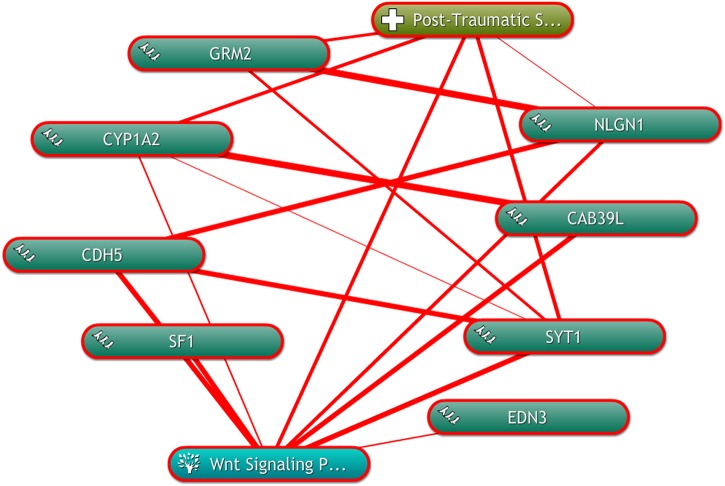
The linear relationship among the “seed” genes, and the Wnt signaling pathway with PTSD by using COREMINE. Three “seed” genes were associated with the Wnt signaling pathway and PTSD. The thicker the line, the closer the connection between the two ends.

To further depict the relationship among the “seed” genes, and the Wnt signaling pathway with PTSD, text mining was conducted using COREMINE. Co-occurrence analysis of the literature was performed using “post-traumatic stress disorder,” “Wnt signaling Pathway,” and “gene symbols” as search terms. Eight genes (GRM2, CYP1A2, CDH5, SF1, EDN3, SYT1, CAB39L, and NLGN1) were identified in the text-mining searches, as shown in Figure 5. We found that seven genes of eight (CYP1A2, CDH5, SF1, EDN3, SYT1, CAB39L, and NLGN1) were correlated with the Wnt signaling pathway. Four genes (GRM2, CYP1A2, SYT1, and NLGN1) were related to PTSD. Moreover, we found that CYP1A2, SYT1, and NLGN1 were associated with the Wnt signaling pathway and PTSD.

### FST, TST, and OFT

**FIGURE 6:**
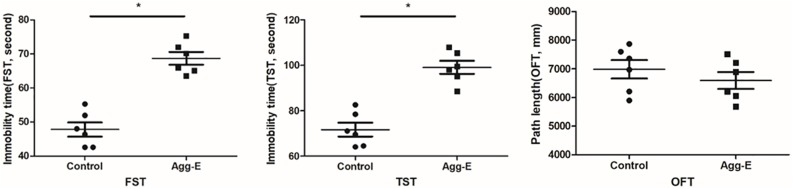
FST and TST immobility time in animal testing and path length of OFT. Results were shown as mean ± SD, ^∗^*P* < 0.05.

Finally, behavioral evaluations were conducted by FST, TST and OFT. It showed that 10 days aggressor exposure induced a significant increase in immobility time. And there was no significant difference on path length of OFT (Figure 6).

## Discussion

Post-traumatic stress disorder is a long term, maladaptive stress condition, which is characterized by various symptoms, e.g., fear, depression, avoidance behavior, and impaired hyperarousal (Chitrala et al., 2016). It presents a considerable economic and health burden for families, communities and countries. The occurrence and development of PTSD are complicated. However, the underlying pathogenesis of PTSD is not fully understood. Thus, in this present study, we tried to identify important contributors and elucidate possible molecular mechanism related to PTSD.

Currently, with the development of high-throughput technology, microarrays and next generation sequencing combined with bioinformatics analysis generated numerous datasets including mRNA, miRNA, and lncRNA expression profile. Millions of genes were detected and widely used to predict potential biomarker of PTSD. In this study, we retrieved dataset of mRNA expression microarrays from GEO, and used bioinformatics analysis to identify key biomarkers and associated pathways related to PTSD. A total of 1004 DEGs (583 up-regulated and 421 down-regulated) were found. Using functional annotations and enrichment analysis, we found the function of these DEGs were closely related to the Wnt signaling pathway. By constructing the PPI network and module analysis, a number of “seed” genes were identified. In order to further understand whether these genes were altered in stress-exposed mice exhibiting PTSD-like features, we verified these genes in experimental study by qRT-PCR. The result indicated that these genes obtained from the PPI network involved in PTSD. So we posit that these genes may be served as potential biomarkers of PTSD. And the FST and TST results showed that Agg-E group had a significant increase in immobility time, which suggested that stress exposure can induced depressive-behavior.

In text mining network, three genes (CYP1A2, SYT1, and NLGN1) were found to be associated with the Wnt signaling pathway. We reasoned that the altered genes affecting PTSD might work through the Wnt signaling pathway. It showed that these genes may play key roles in PTSD via the Wnt signaling pathway. This finding was supported by previous study (Vidal et al., 2018), which suggested that the Wnt signaling pathway plays a significant role in neurogenesis and the maturation of hippocampal neurons. Several genetic studies (Inkster et al., 2010; Matrisciano et al., 2011; Wilkinson et al., 2011; Vidal et al., 2018) have reported that the Wnt receptor as well as the signaling pathway downstream were involved in the stress process. Under acute and chronic stress condition, the protein level of secreted glycoprotein Dickkopf-1 (Dkk-1), an inhibitor of the canonical Wnt pathway, showed a significantly higher than that of the controls (Matrisciano et al., 2011). While the expression of disheveled-2 (DVL2), an important protein of the Wnt pathway in nucleus accumbens (NAc) was decreased in chronic social defeat stress models (Wilkinson et al., 2011). Given this evidence, we further argue that the Wnt pathway is critical in pathogenesis of PTSD. The genesis of PTSD is an extremely complex process during which many genetic and epigenetic modifications of driving genes occur. CYP1A2 is concerned with coding a member of the cytochrome P450 enzymes. It is well studied in treating depressive disorder (Hofer et al., 2013). And SYT1 is a membrane protein, a critical mediator for membrane fusion during the neurotransmitter release induced by Ca^2+^. It can influences synaptic plasticity via the regulation of neurotransmitter release, thus further influencing learning and memory (Zhang et al., 2015). However, no prior reports regarding SYT1 directly associated with PTSD. Further experimental and functional studies are warranted to explore the functional roles of SYT1 related to PTSD. NLGN1, localized at excitatory synapses, plays a critical role in mediating the formation and remodeling of synapses. Previous study (Zhang et al., 2015) found that NLGN1 involved in learning and memory function was closely associated with PTSD. The variation of NLGN1 may lead to higher risk to develop PTSD. In light of our experimental results, we further surmised that these genes may play a positive role on PTSD.

In summary, by using bioinformatics analysis, experimental verification, and text mining, we found that several genes and relevant pathway may represent key mechanisms involved in the development of PTSD. However, these findings require verification in future experimental studies.

## Author Contributions

YB carried out the design of the study, made the data acquisition, and prepared manuscript with LY. MZ, ZL, and YX conducted the data analysis. MZ, YX, and GZ performed the statistical analysis. WL and LZ provided the several suggestions for manuscript revision.

## Conflict of Interest Statement

The authors declare that the research was conducted in the absence of any commercial or financial relationships that could be construed as a potential conflict of interest.

## Footnotes

1 https://david.ncifcrf.gov/

2 https://string-db.org/

3 http://www.coremine.com/medical/
